# Purification and Characterization of a New Alginate Lyase from Marine Bacterium *Vibrio* sp. SY08

**DOI:** 10.3390/md15010001

**Published:** 2016-12-23

**Authors:** Shangyong Li, Linna Wang, Jianhua Hao, Mengxin Xing, Jingjing Sun, Mi Sun

**Affiliations:** 1 Key Laboratory of Sustainable Development of Marine Fisheries, Ministry of Agriculture, Yellow Sea Fisheries Research Institute, Chinese Academy of Fishery Sciences, 106 Nanjing Road, Qingdao 266071, China; lshywln@163.com (S.L.); wlnwfllsy@163.com (L.W.); 15865506157@126.com (M.X.); sunjj@ysfri.ac.cn (J.S.); 2 Laboratory for Marine Drugs and Bioproducts, Qingdao National Laboratory for Marine Science and Technology, Qingdao 266237, China

**Keywords:** alginate lyase, thermostability, unsaturated alginate disaccharides, *Vibrio* sp.

## Abstract

Unsaturated alginate disaccharides (UADs), enzymatically derived from the degradation of alginate polymers, are considered powerful antioxidants. In this study, a new high UAD-producing alginate lyase, AlySY08, has been purified from the marine bacterium *Vibrio* sp. SY08. AlySY08, with a molecular weight of about 33 kDa and a specific activity of 1070.2 U/mg, showed the highest activity at 40 °C in phosphate buffer at pH 7.6. The enzyme was stable over a broad pH range (6.0–9.0) and retained about 75% activity after incubation at 40 °C for 2 h. Moreover, the enzyme was active in the absence of salt ions and its activity was enhanced by the addition of NaCl and KCl. AlySY08 resulted in an endo-type alginate lyase that degrades both polyM and polyG blocks, yielding UADs as the main product (81.4% of total products). All these features made AlySY08 a promising candidate for industrial applications in the production of antioxidants from alginate polysaccharides.

## 1. Introduction

Alginate is a linear hetero-polyuronic acid polymer, composed of β-d-mannuronate (M) and its C5 epimer α-l-guluronate (G), which can be arranged as polyM blocks, polyG blocks, and alternating or random polyMG blocks [1,2,3]. It is the most abundant carbohydrate in brown algae and approximately 30,000 tons of alginate are produced worldwide annually [3,4,5]. The alginate polymer is a commercially valuable polysaccharide widely used in the food and pharmaceutical industries mainly due to its high viscosity and gelling properties [1,3,6].

Alginate lyases catalyze the depolymerization of alginate through a β-elimination reaction between uronic acids in the linear polymer, thus producing unsaturated alginate oligosaccharides (UAOs) with double bonds between C4 and C5 at the non-reducing ends [7,8]. UAOs are endowed with an excellent antioxidant activity, superior to ascorbic acid in lipid oxidation treatment [9,10]. Their antioxidant activity is dependent on the conjugated alkene acid structure occurring in UAOs from the enzymatic depolymerization of alginate [9]. Neither acid hydrolysis of alginate nor alginate monosaccharides present conjugated alkene acid structures, thus exhibiting a lower antioxidant activity in comparison with UAOs [11,12,13]. Moreover, the antioxidant activity is inversely related to the molecular weight of UAOs: the smaller the size of the UAOs, the higher the antioxidant activity [14]. As the minimum unit endowed with the peculiar antioxidant structure, the unsaturated alginate disaccharides (UADs) are thought to be the best antioxidants [9].

Thus far, hundreds of alginate lyases have been isolated and characterized from marine microorganisms, brown seaweeds and mollusks [7,8]. According to their action mechanism, alginate lyases are generally classified into endo- or exo-lytic enzymes [15,16]. The product of exo-type alginate lyases consists of alginate monosaccharides, while the main product of endo-type alginate lyases is a mixture of unsaturated alginate disaccharides, trisaccharides and tetrasaccharides [16,17]. High levels of UADs in the product are beneficial for their antioxidant activity. However, UADs are generally present in low proportions (less than 50% of the total) in the products of most alginate lyases, except for AlyL2 from *Agarivorans* sp. L11 [18,19,20,21,22,23,24,25,26,27]. Although the full-length alginate lyase, AlyL2-FL, is able to produce a high ratio of UADs (64.6% of the total product), the activity strictly depends on the NaCl concentration [22]. The desalting of UAOs, especially for UADs, is very difficult and represents an important limitation on UAD production and application [28]. Therefore, the discovery of new alginate lyases yielding high levels of UADs is of the utmost importance.

Here, we report the isolation and characterization of a new endo-type alginate lyase, AlySY08, from the marine bacterium *Vibrio* sp. SY08. AlySY08 is an enzyme yielding UADs as the main product (81.4% of the total) and its activity is independent of NaCl.

## 2. Results and Discussion

### 2.1. Isolation and Identification of Strain SY08

**Figure 1:**
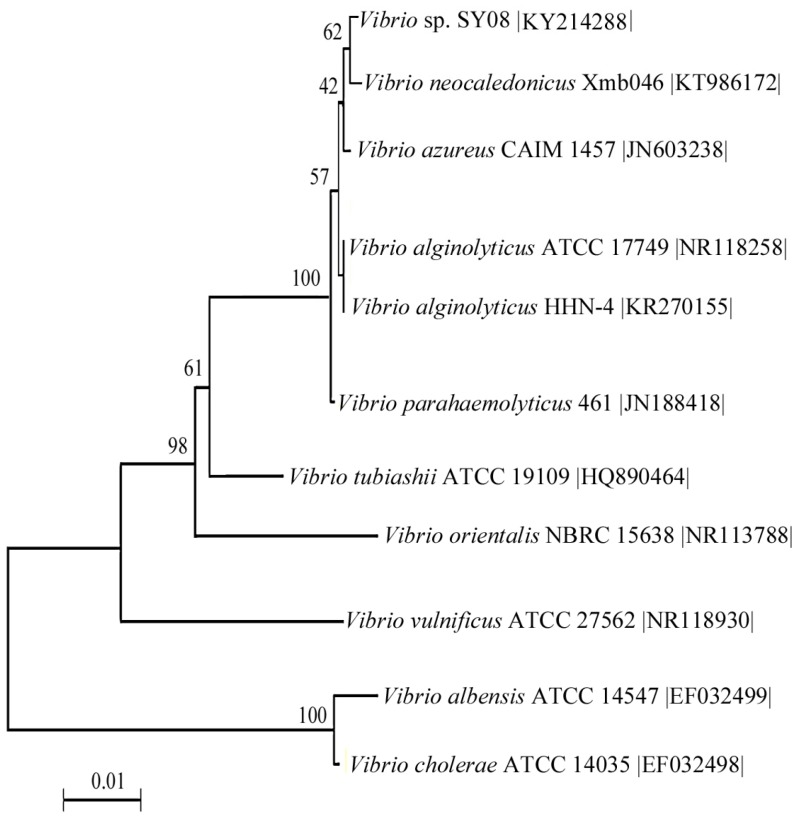
Phylogenetic tree of strain SY08 and related bacteria. The tree ID is based on a maximum parsimony analysis of the 16S rDNA sequences. The obtained 16S rDNA sequence was searched for and aligned by using the BLASTn and ClustalX programs, respectively. The phylogenetic tree was obtained by using MEGA 4.0 software.

Through screening the sole carbon source, we detected 29 strains with alginate lyase activities in their culture supernatants. Among these strains, SY08 showed the highest activity and was selected it for further research. A 1411 bp fragment of the 16S rDNA gene of the strain SY08 (Genbank accession number: KY214288) was cloned and sequenced. The alignment of 16S rDNA gene sequences revealed that strain SY08 was 99% identical to the *Vibrio* strain. According to the phylogenetic position of its 16S rDNA (Figure 1), SY08 was assigned to the genus *Vibrio* and named *Vibrio* sp. SY08.

### 2.2. Purification and Biochemical Characterization of AlySY08

**Figure 2:**
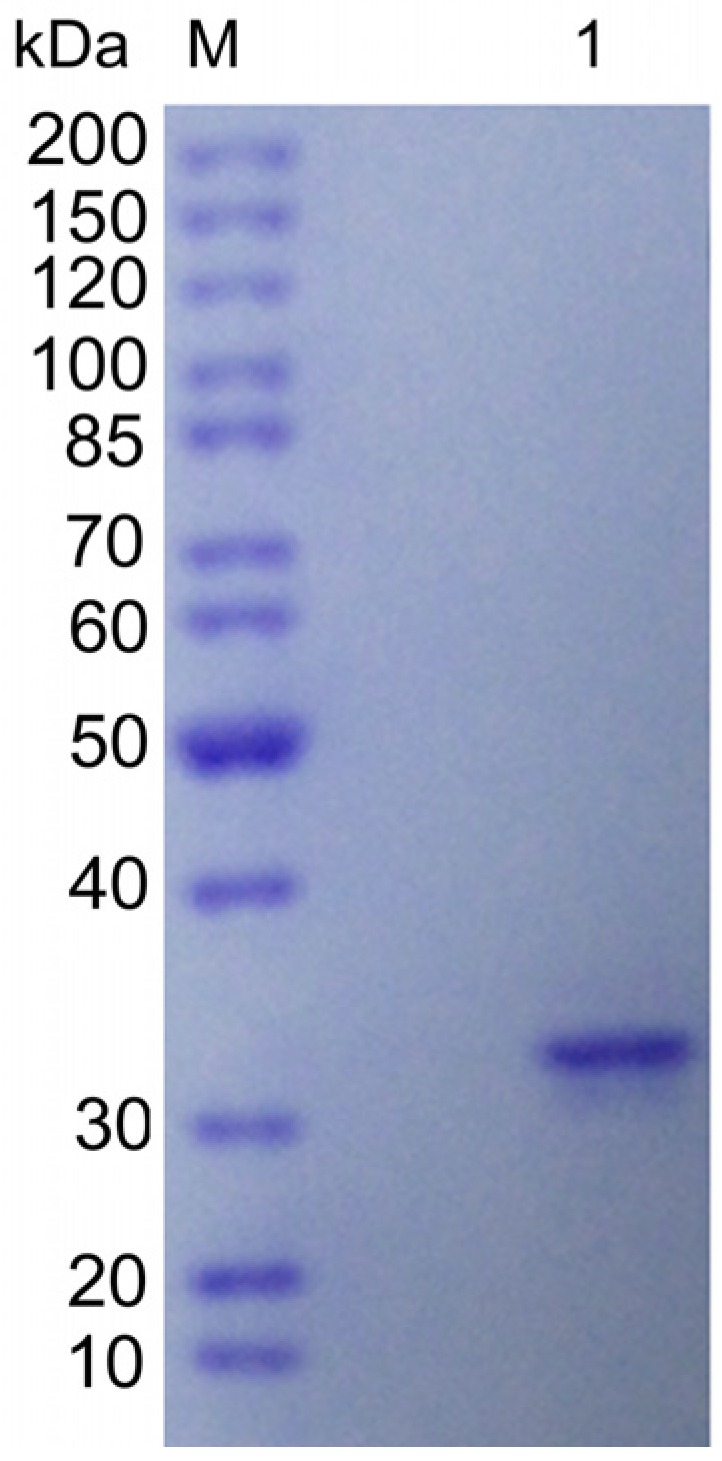
SDS-PAGE analysis of purified AlySY08. The purified AlySY08 was resolved by 10% acrylamide (*w*/*v*) SDS-PAGE followed by staining with Coomassie Blue G-250. Lane M, molecular weight markers; Lane 1, purified AlySY08.

The enzyme was purified to 13.1-fold homogeneity with a specific activity of 1070.2 U/mg. The purification was achieved through ammonia sulfate precipitation and only one hydrophobic interaction chromatography step (Table S1). The activity recovery of alginate lyase was 43.6%, and about 2.1 mg of purified AlySY08 could be obtained from 1 L of strain SY08 culture supernatant. The purity of AlySY08 was evaluated by SDS-PAGE, along with the molecular weight, estimated to be approximately 33 kDa (Figure 2).

**Figure 3:**
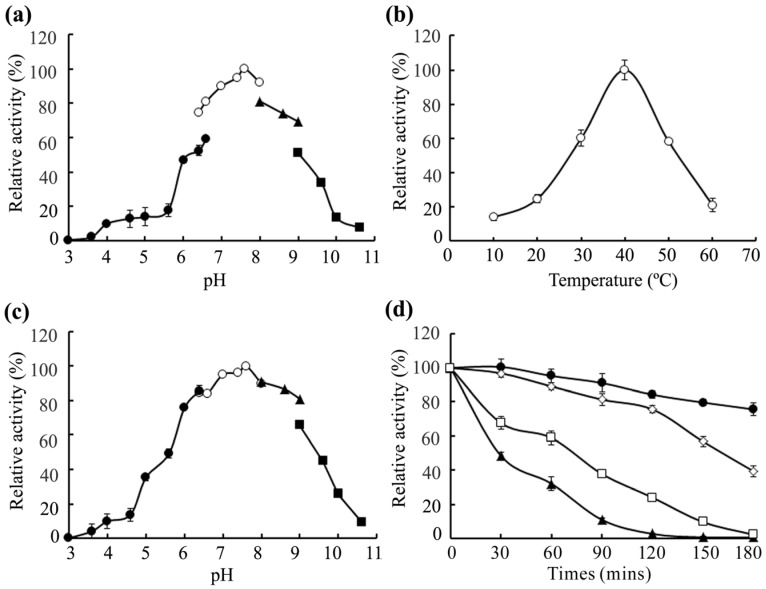
Effects of pH and temperature on the activity and stability of AlySY08. (**a**) The optimum pH for AlySY08 was determined by measuring its activity at 40 °C in 50 mM Na_2_HPO_4_-citric acid buffer (filled circle), 50 mM Na_2_HPO_4_-NaH_2_PO_4_ buffer (open circle), 50 mM Tris-HCl buffer (filled triangle) and 50 mM Gly-NaOH buffer (filled square); (**b**) The optimal temperature for AlySY08 was determined by measuring its activity at various temperatures (10–60 °C); (**c**) pH stability of AlySY08. The residual activity was measured at 40 °C in 50 mM phosphate buffer (pH 7.6) after incubation in the buffers reported above at 4 °C for 6 h; (**d**) Thermostability of AlySY08. The enzyme was incubated at 30 °C (filled circle), 40 °C (open rhombus), 45 °C (open square) and 50 °C (filled triangle) for various times. The residual activity was then determined at 40 °C. The activity of control (100% relative activity) is 12.6 U/mL.

AlySY08 showed the highest activity in phosphate buffer at pH 7.6 (Figure 3a), remaining stable in a range of pH 6.0–9.0 (Figure 3c). The optimal temperature of AlySY08 was 40 °C (Figure 3b). AlySY08 retained ~90% and ~75% of its activity after incubation at 30 °C and 40 °C for 120 min, respectively. It still retained ~40% of its activity after being incubated at 45 °C for 90 min or 50 °C for 40 min (Figure 3d). Because of the long half-life, high temperature stability and low production costs, thermostable enzymes are widely used in industrial applications. As previously reported, alginate lyases from the genera *Pseudoalteromonas*, *Agarivorans*, *Microbulbifer* and most of *Vibrio* are stable only at temperatures below 40 °C for less than 60 min [23,24,27,29,30]. Only few alginate lyases are thermostable, such as AlyV5 from *Vibrio* sp. QY105 which retains 40% of its activity after incubation at 60 °C for 60 min or 90 °C for 10 min [25]; the alginate lyase from *Isoptericola halotolerans* CGMCC 5336 which retains 60% of its activity after incubation at 40 °C for 180 min [18]; and AlyL2-FL from *Agarivorans* sp. L11 which retains 50% of its activity after incubation at 40 °C for 120 min or 50 °C for 70 min [22]. The main products of AlyV5 and the alginate lyase from *I. halotolerans* CGMCC 5336 consist of a mixture of hard-to-separate disaccharides, trisaccharides, tetrasaccharides and pentasaccharides [18,25,31]. In addition, both AlyV5 and AlyL2 are NaCl-dependent enzymes, since very low levels of enzymatic activities have been detected in the absence of NaCl [22,25].

**Figure 4:**
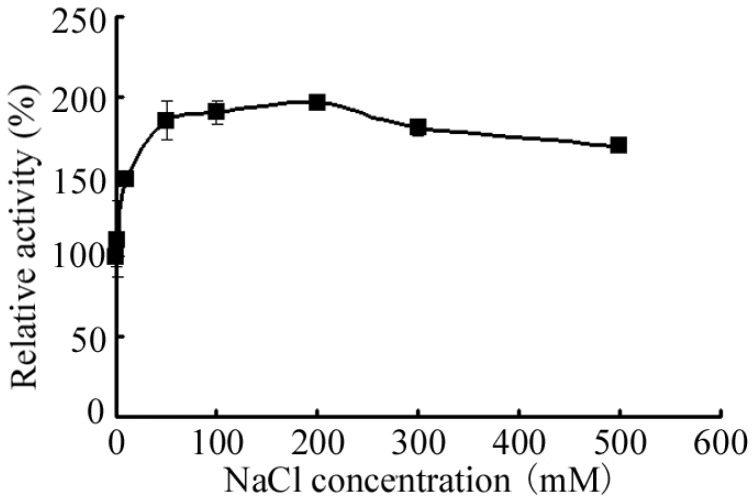
Effect of NaCl on enzymatic activity of AlySY08. The activity of AlySY08 in the absence of NaCl was retained at 100%. All the experiments were conducted in triplicate.

Although the activity of AlySY08 could be enhanced by the addition of NaCl and KCl, the enzyme was still active in the absence of NaCl and KCl (Figure 4 and Table S2). In more detail, Cu^2+^, Zn^2+^, Mn^2+^, Al^3+^ and Fe^3+^ inhibited the activity of AlySY08, while Mg^2+^ and Ca^2+^ enhanced its activity. Both of the chelating agents (EDTA and SDS) and the reducing agent (2-Mercaptoethanol) significantly inhibited the activity of AlySY08 (Table S2).

### 2.3. Substrate Specificity and Kinetic Parameters of AlySY08

The substrate specificity of AlySY08 was evaluated by using alginate, polyG blocks and polyM blocks as substrates. Among the assayed polymeric substrates, AlySY08 was revealed to be more active towards polyG blocks with respect to polyM blocks and alginate (Table 1).

The activity towards sodium alginate was determined as the 100% relative activity. All the experiments were conducted in triplicate. The data are expressed as the mean ± SD.

The kinetic parameters of AlySY08 relative to the cleavage of various alginate polymers are also shown in Table 1. The apparent *K*_m_ of AlySY08 against sodium alginate, polyG blocks, and polyM blocks was 0.36, 0.34 and 0.85 mg/mL, respectively. The *V*max of AlySY08 against alginate, polyG blocks, and polyM blocks was 1183.7, 1255.5 and 512.9 U/mg, respectively. Both the specific activity and kinetic parameters indicated that AlySY08 is active towards both polyG and polyM blocks, but degrades the former more efficiently. As previously reported, most of the alginate lyases are polyM-preferred alginate lyases. Only few alginate lyases are polyG-preferred alginate lyases, including AlyV5 from *Vibrio* sp. QY105 [25], alginate lyase from *Vibrio* sp. 510-64 [28] and alginate lyase from *Streptomyces* sp. ALG-5 [30].

### 2.4. Mode of Enzyme Action and Reaction Products of AlySY08

**Figure 5:**
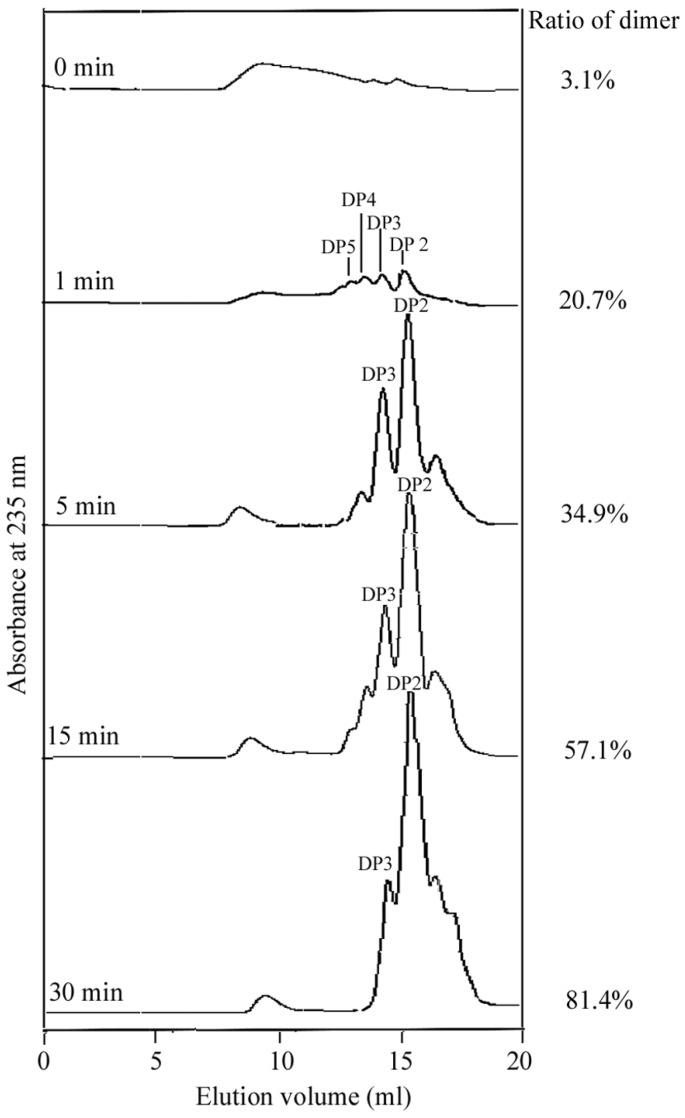
Size-exclusion chromatography of the alginate degradation products by AlySY08. The elution volumes of the dimer (DP2), trimer (DP3), tetramer (DP4), and pentamer (DP5) are 16.1 mL, 14.9 mL, 14.1 mL and 13.7 mL, respectively. The ratios of dimers present in the degradation products were analyzed by the peak integration function on the UNICORN 5.31 software (GE Healthcare, Madison, WI, USA).

The mode of the enzyme action was monitored by size-exclusion chromatography (Figure 5). The rapid degradation of the substrate, the increase in polydispersity and the production of intermediate oligosaccharides suggested that AlySY08 acts in an endo-lytic mode [23]. Moreover, as shown in the viscometric assay (Figure S1), after adding AlySY08, the viscosity of the alginate solution decreased rapidly in first 5 min, but changed little in the following 25 min. The amount of reducing sugar (A235) increased steadily during the whole observation period (Figure S1). All of these findings suggested that the enzyme is an endo-type enzyme.

**Figure 6:**
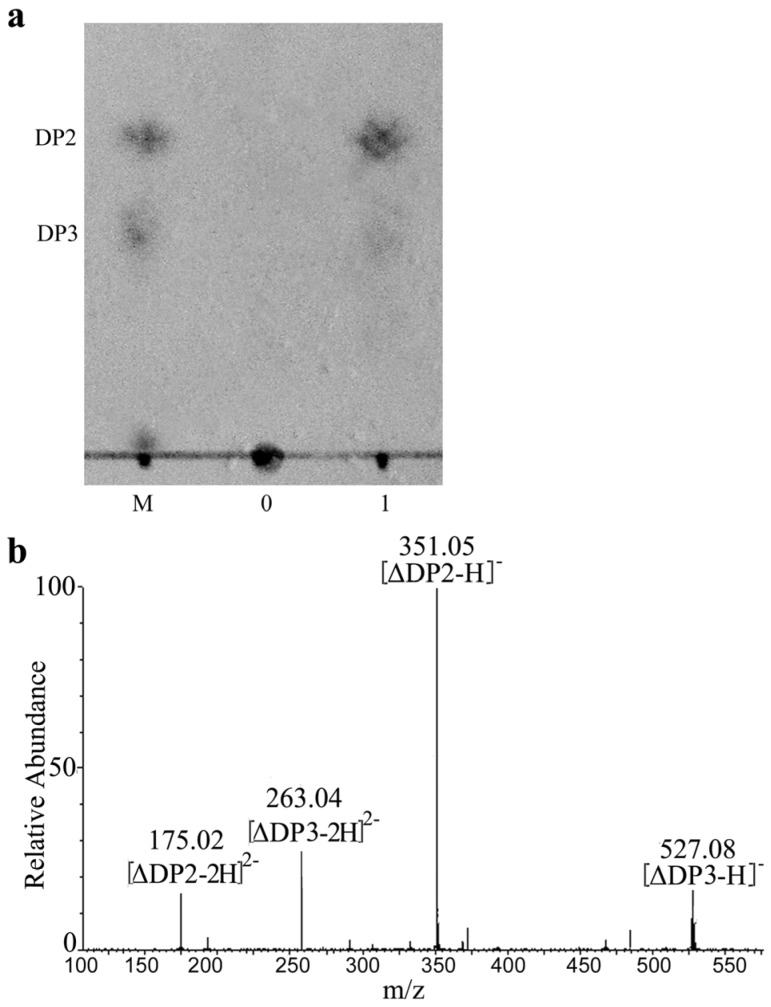
TLC and ESI-MS analysis of the main products of AlySY08. (**a**) TLC analysis. The reaction products were separated on a HPTLC plate with *n*-butanol/formic acid/water (2:1:1, by vol) and visualized with a diphenylamine/aniline/phosphate reagent. Lane M: standard UAOs mixture, disaccharide (DP2) and trisaccharide (DP3); Lane 0 sodium alginate; Lane 1 reaction products; (**b**) ESI-MS analysis of the main products by AlySY08.

Degradation products obtained from the most common endo-type alginate lyases are usually determined by TLC [7], clearly indicating that the main products are a mixture of alginate penta-, tetra-, tri- and di-saccharides [7,24,25,26,27,29]. However, when we analyzed the reaction products of AlySY08 by TLC, we observed only one spot on the TLC plate. The migration rate of this spot was in good agreement with the UAD marker (Figure 6a). When the reaction products of AlySY08 were analyzed by size-exclusion chromatography with a Superdex peptide 10/300 column, UADs were revealed as the main product (81.4% of the total product). The reaction products were further identified by negative ESI-MS. As reported in Figure 6b, the main peaks at 351.05 *m*/*z* [∆DP2-H]^−^ and 175.02 *m*/*z* [∆DP2-2H]^2−^ corresponded with the UAD [26]. All of these results indicated that the main product of AlySY08 consists of UADs.

If compared with other alginate lyases, AlySY08 shows the highest UAD-yielding levels (Table 2). A high ratio of UADs in the mixture of products is beneficial for antioxidant activity. Recently, depolymerization products of many other alginate lyases were also characterized by using size-exclusion chromatography (Table 2). Although the full-length alginate lyase AlyL2-FL produces a high ratio of UADs (64.6% of the total), its activity is strictly dependent on the NaCl concentration [22]. The concentration of UADs produced by the mutated alginate lyase MJ3-Arg236Ala, from *Sphingomonas* sp. MJ-3 is about 80.6% towards polyM blocks, about 47.1% towards polyMG blocks and only 37.5% towards alginate [17].

## 3. Materials and Methods

### 3.1. Isolation and Identification of Strain SY08

Decayed brown seaweed samples were collected from the coastal zone of Jiaozhou Bay, Qingdao, China. They were immersed, diluted, and spread on the fermentation medium agar plate (5 g sodium alginate, 30 g NaCl, 0.01 g MgSO_4_, 2 g (NH_4_)_2_SO_4_, 0.02 g FeSO_4_, 3 g K_2_HPO_4_, 7 g KH_2_PO_4_, 15 g agar in 1 L distilled water, pH 6.5). The plates were incubated at 25 °C for two days to form the detectable colonies. At least 300 strains were inoculated into selective medium without agar and assayed for alginate lyase activity in the culture supernatant. The 16S rDNA gene was amplified according to the method described by Wang et al. 2013 [25]. The obtained 16S rDNA gene sequence was searched and aligned with its closely related sequences retrieved from GenBank using the BLASTn (National Center of Biotechnology Information, Bethesda, MD, USA) and Clustal W programs (Conway Institute UCD Dublin, Dublin, Ireland). Multiple sequence alignments were obtained using ClustalX 1.83 (Conway Institute UCD Dublin, Dublin, Ireland), and the phylogenetic tree was constructed with the MEGA 4.0 software (Biodesign Institute, Arizona State University, Tempe, AZ, USA).

### 3.2. Purification of AlySY08

Strain SY08 was inoculated in 1 L selective medium and cultured at 25 °C for 48 h with shaking at 120 rpm. The culture supernatant of strain SY08 was obtained by centrifugation at 12,000× *g* for 10 min. Then, ammonium sulfate was added to a final saturation of 40% and incubated for 2 h. After centrifugation (12,000× *g*, 30 min), the supernatant was loaded onto a Phenyl-Sepharose column (1.6 cm × 20 cm) equilibrated with 50 mM phosphate buffer (pH 7.6), then eluted with a linear gradient of (NH_4_)_2_SO_4_ (1.5–0 M, 100 mL) at a flow rate of 1 mL/min. All steps of enzyme purification were carried out at 4 °C. The active fractions were stored at −20 °C. The molecular weight of purified AlySY08 was determined by SDS-PAGE. The protein concentration was determined by the Bradford method with bovine serum albumin (BSA) as standard.

### 3.3. Alginate Lyase Activity Assay

Alginate lyase activity was measured as the increase in the absorbance at 235 nm. In brief, the enzymatic reaction was conducted with 100 μL of enzyme solution and 900 μL of substrate solution (0.3% (*w*/*v*) alginate, 50 mM phosphate buffer, pH 7.6) at 40 °C for 10 min. One unit (U) was defined as the amount of the enzyme required to increase by 0.1 the absorbance at 235 nm per minute. For the study of substrate specificities, polyM blocks and polyG blocks were used as substrates in the same conditions.

### 3.4. Effects of Temperature, pH, Metal Ions and Chelators

The optimum pH of the enzyme was determined by measuring its activity in different buffers at 40 °C for 10 min [23]. Its optimal temperature was determined by measuring its activity in a range of 10–60 °C in 50 mM phosphate buffer (pH 7.6). To determine pH stability of AlySY08, the residual activity was measured after enzyme was incubated in different buffers for 6 h at 4 °C. The thermostability of the enzyme was evaluated by measuring the residual activity after the enzyme in 50 mM phosphate buffer (pH 7.6) was incubated at 30 °C, 40 °C, 45 °C and 50 °C for different times (0, 30, 60, 90, 120, 150, 180 min). The effects of metal ions and chelators on AlySY08 activity were examined by monitoring the enzymatic activity in the presence of various cation ions or chelators.

### 3.5. Enzymatic Kinetic Parameters Assay

To measure the kinetic parameters of AlySY08, 10 different concentrations (ranging from 0.1 to 8 mg/mL) of alginate, polyM blocks and polyG blocks in 50 mM phosphate buffer (pH 7.6) were incubated at 40 °C for 10 min. AlySY08 was then added to a final concentration of 10 nM, and incubated at 40 °C for 3 min. The *K*_m_ and *V*_max_ values were calculated by double-reciprocal plots of Lineweaver and Burk [27].

### 3.6. Analysis of Reaction Mode and Products

The reaction mixture containing 0.5 mL (20 U) of purified AlySY08 and 2 mL of sodium alginate (1 mg/mL) in 50 mM phosphate buffer (pH 7.6) was incubated at 40 °C for 1, 5, 15 or 30 min. Reaction products were analyzed by the fast protein liquid chromatography (FPLC) with a Superdex peptide 10/300 gel filtration column (GE Healthcare, Madison, WI, USA) as previously described by Li et al. 2015 [22,23]. Then, products obtained from the enzymatic action (30 min) of AlySY08 on alginate were further characterized by using thin-layer chromatography (TLC) and negative-ion electrospray ionization mass spectrometry (ESI-MS), as previously reported [26,28]. To further determine its action mode, the viscometric assay was done using as Ostwald viscometer (No. 1; Shibata Scientific Technology LTD., Soka-City, Saitama, Japan) [32]. Briefly, mixtures of 5 mL AlySY08 (5 U/mL) and 50 mL sodium alginate (2 g/L in 20 mM phosphate buffer, pH 7.6) were incubated at 40 °C for up to 30 min. An aliquot of enzymatic product (0.5 mL) was taken out at different times (1, 5, 10, 15 and 30 min) in order to determine the viscosity and degradation products.

## 4. Conclusions

In this study, AlySY08, an alginate lyase from the marine bacterium *Vibrio* sp. SY08, was purified and characterized. AlySY08 showed the highest activity at 40 °C in phosphate buffer at pH 7.6. The enzyme was stable over a broad pH range (6.0–9.0) and active in the absence of salt ions. AlySY08 yielded UADs, a promising class of molecules with powerful antioxidant activity, as the main product (81.4% of the total product), thus representing an interesting candidate for industrial applications. Further works will be focused on gene cloning and elucidating the molecular mechanism of action of AlySY08, along with the determination of its three-dimensional structure.

## Figures and Tables

**Table 1:** The substrate specificity and kinetic parameters of AlySY08.

Substrate	Relative Activity (%)	*K*_m_ (mg/mL)	*V*_max_ (U/mg)
Sodium alginate	100.0 ± 0.8	0.36 ± 0.04	1183.7 ± 21.5
PolyG blocks	123.8 ± 2.8	0.34 ± 0.02	1255.5 ± 14.7
PolyM blocks	28.2 ± 1.3	0.85 ± 0.16	512.9 ± 8.3

**Table 2:** Comparison of the main products of AlySY08 with other alginate lyases.

Enzyme	Source	Main Products (DP)	Ratio of Dimer (%) ^a^	Reference
AlySY08	*Vibrio* sp. SY08	2	81.4	This study
AlyL2-FL	*Agarivorans* sp. L11	2–3	64.6	[22]
AlyL2-CM	*Agarivorans* sp. L11	2–3	52.6	[22]
AlyL1	*Agarivorans* sp. L11	2–3	47.3	[23]
MJ3-Arg236Ala	*Sphingomonas* sp. MJ-3	2–5	37.9	[17]
AlgMsp	*Microbulbifer* sp. 6532A	2–5	37.5	[26]
AlyA1	*Zobellia galactanivorans*	2–6	19.0	[24]
Aly5	*Flammeovirga* sp. MY04	2–7	15.7	[19]

^a^ The ratio of dimers was determined by the peak integration function of the UNICORN 5.31 software.

## Supplementary Materials

The following are available online at www.mdpi.com/1660-3397/15/1/1/s1, Table S1: Summary of AlySY08 purification, Table S2: Effect of metal ions, chelators and detergents on the activity of AlySY08, Figure S1: Viscosity reduction during enzymatic degradation of alginate. The initial viscosity of the reaction mixture without enzyme was taken as 100%. Open circles with solid line rate of viscosity reduction; filled circles with dotted line absorbance at 235 nm.
