# Supplementary material for: Purification and Characterization of a New Alginate Lyase from Marine Bacterium Vibrio sp. SY08
Source: Mar Drugs. 2016 Dec 23;15(1):1. doi: 10.3390/md15010001 (PMC5295221; doi:10.3390/md15010001)
Supplement: Supplementary file 1 [file marinedrugs-15-00001-s001.doc]

Supplementary Materials: Purification and Characterization of a New Alginate Lyase from Marine Bacterium *Vibrio* sp. SY08

Shangyong Li, Linna Wang, Jianhua Hao, Mengxin Xing, Jingjing Sun and Mi Sun

**Table S1.** Summary of AlySY08 purification.

| **Purification Step** | **Total Activity (U)** | **Total Protein (mg)** | **Specific Activity (U/mg)** | **Folds** | **Recovery (%)** |
| --- | --- | --- | --- | --- | --- |
| Crude extract | 490 | 4.1 | 119.5 | 1 | 100 |
| (NH_4_)_2_SO_4_ precipitation | 369 | 1.5 | 246.5 | 2.1 | 75.3 |
| Phenyl-Sepharose | 214 | 0.2 | 1070.2 | 13.1 | 43.6 |

Values given are the average of three replications.

**Table S2.** Effect of metal ions, chelators and detergents on the activity of AlySY08.

| **Additives** | **Concentration (mM)** | **Relative Activity (%)** |
| --- | --- | --- |
| Control | -- | 100 ± 4.1 |
| KCl | 1 | 111.3 ± 8.6 |
| LiCl | 1 | 40.9 ± 2.6 |
| NH_4_Cl | 1 | 60.3 ± 6 |
| ZnCl_2_ | 1 | 19.1 ± 0.7 |
| CuCl_2_ | 1 | 14.4 ± 2.4 |
| MnCl_2_ | 1 | 48.6 ± 1.8 |
| CaCl_2_ | 1 | 103.7 ± 2 |
| MgCl_2_ | 1 | 119.8 ± 3.8 |
| FeCl_3_ | 1 | 71.8 ± 0.9 |
| AlCl_3_ | 1 | 61.9 ± 5.1 |
| EDTA | 1 | 57.4 ± 3.8 |
| SDS | 1 | 10.7 ± 2.5 |
| 2-Mercaptoethanol | 1 | 32.5 ± 3.1 |

The data were expressed as mean ± SD, *n* = 3. The activity of control (100% relative activity) is 12.2 U/mL.


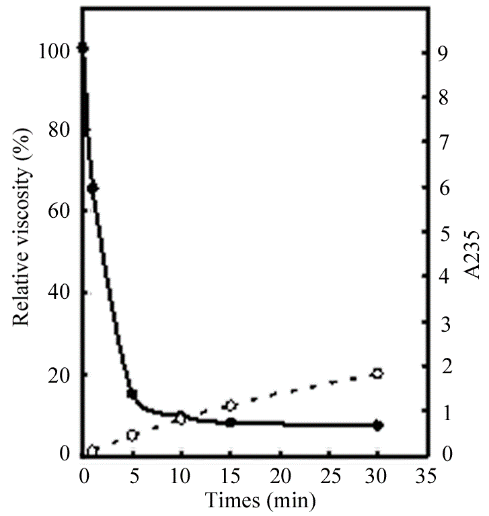


**Figure S1.** Viscosity reduction during enzymatic degradation of alginate. The initial viscosity of the reaction mixture without enzyme was taken as 100%. Open circles with solid line rate of viscosity reduction; filled circles with dotted line absorbance at 235 nm.
